# A Study at Wad Madani, Sudan: Are We Documenting Acute Ankle Fractures Effectively?

**DOI:** 10.7759/cureus.68333

**Published:** 2024-08-31

**Authors:** Ahmed Mohamed, Abubakr Muhammed

**Affiliations:** 1 Department of Orthopaedics, Gezira Centre for Orthopedic Surgery and Traumatology, Wad Madani, SDN; 2 Department of Surgery, University of Gezira, Madani, SDN

**Keywords:** documentation, acute ankle fractures, british orthopaedic association standards, clinical assessment, national standards

## Abstract

Background: Medical records are essential documents that outline a patient's medical history and current health status. It involves maintaining records that include assessments of patient outcomes, care plans, and interventions necessary to meet patient needs. A patient's medical record encompasses details about their condition, as documented by healthcare professionals, including clinical assessments, evaluations, and professional opinions related to the delivery of care.

Methods: This retrospective study aimed to evaluate the adequacy of our documentation for acute ankle fractures in accordance with the British Orthopaedic Association Standards for Trauma and Orthopaedics (BOAST) guidelines, encompassing a total of 41 cases. The research was conducted at the Gezira Center for Orthopedic Surgery and Traumatology (GCOST) in Wad Madani, Sudan, from May 12 to July 12, 2022.

Results: Of the 41 recorded notes for acute ankle fractures, 26 (63.4%) were documented by medical officers and 15 (36.6%) by orthopaedic trainees. Most fractures (25 cases, 61%) occurred in individuals aged 18-40 years, and the gender distribution showed that males accounted for most fractures, with 29 cases (70.7%). Additionally, all patients (100%) had a documented cause of injury. Skin integrity was noted in 38 patients (92.7%). Vascular examination was documented in 18 patients (43.9%), while neurological examination was recorded in 16 patients (39%).

Conclusion: Although the cause of ankle fractures was reported in all patients, the neurovascular examination was insufficiently documented, compromising patient care and failing to meet national standards, as highlighted in our study. We recommend implementing the BOAST guidelines to ensure proper documentation and essential assessments.

## Introduction

Medical documentation is the process of recording patient outcomes, care plans, and the interventions necessary to address patient needs. It encompasses a comprehensive account of the patient's condition as reported by healthcare professionals, including clinical assessments, evaluations, and professional judgments related to the delivery of patient care [1].

Medical documentation is a crucial component of patient care, providing detailed information and evidence for patients [2]. It involves recording what occurred, and when it exactly occurred, including medical practices by healthcare professionals [3,4].

The documentation of a patient's medical record is crucial for the continuity of patient care, research, legal defense, and reimbursement, and for the improvement of communication among healthcare providers [5]. Medical documentation must be patient-focused, accurate, relevant, clear, permanent, confidential, and timely, regardless of whether it is electronic or paper-based [6]. Nonetheless, insufficient and incomplete medical documentation leads to a breakdown in communication, which, in turn, impacts medico-legal issues and patient management [7].

The National Institute for Health and Care Excellence (NICE) has released comprehensive guidelines outlining the process of evaluating, monitoring, and treating ankle fractures [8,9]. Although these guidelines stress the critical importance of accurate and thorough documentation, it may not always be a priority in emergencies. There is concern that the documentation for trauma patients and individuals with non-complex fractures, in particular, is not always handled as well as it should be [9,10].

This study aimed to evaluate the adequacy of our documentation for acute ankle fractures per the British Orthopaedic Association Standards for Trauma and Orthopaedics (BOAST) guidelines.

## Materials and methods

Study design

This retrospective study was conducted at Al Gezira Center for Orthopedic Surgery and Traumatology (GCOST) in Wad Madani, Sudan, a major orthopedic facility serving a diverse population.

Study population

The study included patients diagnosed with acute ankle fractures between May 12 to July 12, 2022. This study focused on adult patients over 18 years of age who presented with closed malleolar fractures. Cases involving skeletally immature patients were excluded from the review to ensure a focus on a specific patient group.

Data collection

A detailed review of patient admission files was conducted within the specified period to identify cases of ankle fractures within the specified period. The documentation was meticulously analyzed to ensure an understanding of our documentation practices and areas needing improvement. The variables collected included patient age, gender, and documentation practices.

Documentation assessment

The effectiveness of documentation was assessed based on the guidelines set by the BOAST. The presence and quality of documentation in orthopedic clerking were analyzed based on six specific data parameters. These parameters aimed to ensure precise and comprehensive clerking documentation that meets national standards. The parameters included (1) the case of injury; (2) the condition of the skin at presentation; (3) vascular and circulation status; (4) a neurological examination at presentation, including sensory and motor assessments; (5) a neurovascular examination following reduction; and (6) an overall record of clinical findings at presentation.

Data analysis

The data were coded and cleaned in Microsoft Excel (Microsoft Corporation, Redmond, WA).

Ethical considerations

Ethical approval was obtained from the Institutional Review Board at GCOST, with approval number SUD23/GCOST-23. All personal and clinical data were anonymized before analysis to ensure privacy and confidentiality.

## Results

Out of the 41 recorded notes for acute ankle fractures that were examined in the study, 26 (63.4%) were documented by medical officers, while 15 (36.6%) were documented by orthopedic residents (Table 1). The distribution emphasizes the vital contribution of medical officers in the initial documentation and treatment of ankle fractures, which is essential for ensuring effective patient care. Out of the total cases of acute ankle fractures analyzed, the majority (25 cases, 61%) occurred in individuals aged 18-40 years. The gender distribution revealed that most fractures were in males, accounting for 29 cases (70.7%) (Table 2).

**Table 1:** Overview of the personnel who conducted the examination and documented the notes

Personnel	Number (n)	Percentage (%)
Resident trainee	15	36.6
Medical officer	26	63.4

**Table 2:** Overview of age and gender distribution

Age group (years)	Number (n)	Percentage (%)
18-40	25	61
41-60	15	36.6
>60	1	2.4
Gender	Number (n)	Percentage (%)
Male	29	70.7
Female	12	29.3

All patients (100%) had a documented case of injury. The condition of the skin, particularly its integrity, was documented in 38 patients, representing 92.7% of the cases. Vascular examination, which assesses the blood flow to the affected area, was documented in 18 patients, accounting for 43.9% of the cases. Similarly, a neurological examination was documented in 16 (39%) patients.

After the reduction of 15 patients with ankle fractures, neurovascular examinations were documented for 13 patients, accounting for 86.7%. Table 3 demonstrates a thorough evaluation of the compliance of documentation standards with the BOAST guidelines. This emphasizes adherence to the standards and identifies areas that need improvement.

**Table 3:** An overview of the analysis conducted on the documentation of ankle fracture admissions

Criteria	Documentation (n)	Documentation (%)
The mechanism of injury should be precisely documented at presentation	41	100
Skin integrity should be precisely documented at the presentation	38	92.7
Vascular/circulation status should be documented at presentation	18	43.9
Neurological examination should be documented at presentation	16	39
Following reduction, the neurovascular examination must be repeated and documented	13	86.7
Overall documentation of clinical findings at presentation	15	36.6

## Discussion

The precise implications of inadequate documentation have not been thoroughly explored in the existing body of literature. Nevertheless, the initial process of recording and documenting ankle fractures is crucial because it enables clinicians to establish a fundamental neurovascular condition and make informed decisions regarding treatment [11]. Neurovascular dysfunction can serve as an indication for immediate surgical intervention, and a distinct neurological injury can offer insight into the pattern and severity of the injury [12]. Serial examinations are useful for monitoring neurovascular changes and identifying any improvement or deterioration. In patients who undergo surgery, assessing the neurovascular status before the operation helps distinguish between injuries caused by medical intervention and those resulting from the initial injury mechanism [13]. BOAST guidelines recommend meticulous documentation of clinical findings, emphasizing its importance as a medicolegal matter. Research also indicates that inconsistencies between preoperative observations and documentation can lead to negative outcomes [14].

A neurovascular assessment must be performed before and after ankle manipulation. If there are concerns about vascular compromise or skin tenting, urgent reduction should be attempted to restore vascular flow, followed by reassessment. A handheld Doppler is a quick, noninvasive tool for assessing vascular flow. Additionally, neurological assessment should cover motor and sensory functions [15]. Failure to promptly identify neurovascular impairment can lead to the amputation of a limb or, in severe cases, the patient's demise [16].

Our study found that the documentation standards for ankle fractures at our center do not adhere to the BOAST guidelines. The primary issue identified was an insufficiency in the documentation of neurovascular observations. It is possible that the physicians conducted these assessments but failed to record them. This oversight may be attributed to the high patient volume and workload at our center, which is one of the busiest centers in Sudan. Our hospital serves not only the local population but also numerous surrounding towns and villages.

To tackle this issue, we suggest adopting a uniform documentation procedure that follows the BOAST guidelines. This protocol should prioritize the significance of thorough neurovascular assessments. Moreover, it should incorporate comprehensive checklists to ensure the recording of all crucial parameters and regular audits to oversee compliance and identify opportunities for enhancement. Additionally, the educational intervention significantly enhances documentation standards, leading to better patient care, as demonstrated in a study by Snober et al. [11]. That study found that, following the educational intervention, the adequacy of documentation improved from 86% to 100%.

However, it is important to acknowledge the limitations of our study. The sample size was relatively small, which may affect the generalizability of our findings. Additionally, the study was conducted at a single center, which may limit the applicability of the results to other institutions.

## Conclusions

The insufficient documentation of neurovascular observations in cases of ankle fractures presents substantial hazards to patient care, failing to meet national standards. Thorough documentation is essential for guiding treatment decisions and preventing serious consequences such as limb amputation. We recommend implementing the BOAST guidelines, which prioritize the importance of accurate documentation and help ensure the conduct of critical neurovascular assessments.

Enforcing BOAST guidelines requires providing training to staff members and conducting frequent audits to guarantee adherence. Placing documentation as a top priority will improve the quality of patient care and reduce the occurrence of preventable complications in cases of ankle fractures.
